# Supplementary material for: Gap Junction-Mediated Intercellular Communication of cAMP Prevents CDDP-Induced Ototoxicity via cAMP/PKA/CREB Pathway
Source: Int J Mol Sci. 2021 Jun 13;22(12):6327. doi: 10.3390/ijms22126327 (PMC8231879; doi:10.3390/ijms22126327)
Supplement: Supplementary file 1 [file ijms-22-06327-s001.zip › ijms-1240392-supplementary.pdf]

Gap junction-mediated intercellular communication of cAMP prevents CDDP-induced ototoxicity via cAMP/PKA/CREB pathway

Yeon Ju Kim [1], Jin-Sol Lee [2], Hantai Kim [1], Jeong Hun Jang [1] and Yun-Hoon Choung [1,2]

,

[1] Department of Otolaryngology, Ajou University School of Medicine, Suwon, 16499, Republic of Korea

[2] Department of Medical Sciences, Ajou University Graduate School of Medicine, Suwon, 16499, Republic of Korea

Correspondence

Yun-Hoon Choung, MD, DDS, PhD

Department of Otolaryngology, Ajou University School of Medicine, San 5, Wonchon-dong, Yeongtong-gu, Suwon 443-721, Republic of Korea.

Tel: +82-31-219-5263; Fax: +82-31-219-5264; E-mail address: yhc@ajou.ac.kr

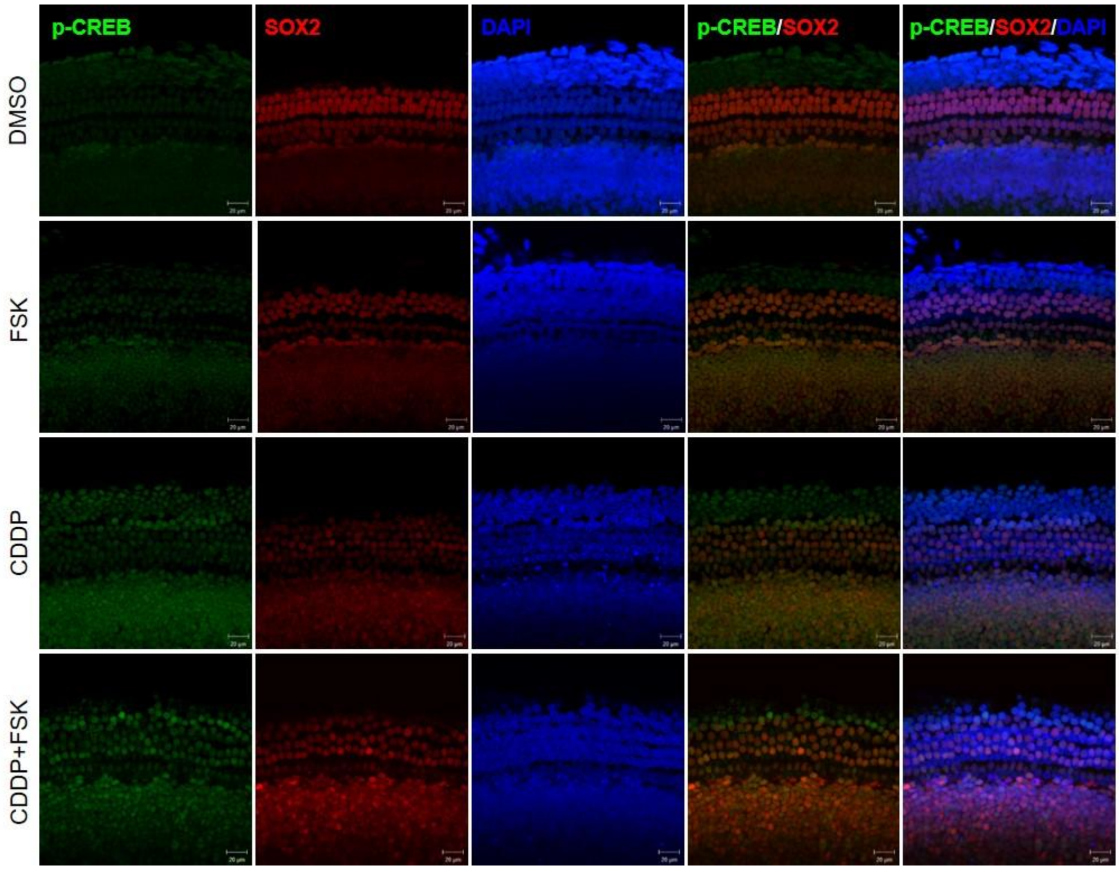

Supplementary Figure 1. Expression of phosphorylated CREB in the organ of Corti. Representative confocal images of p-CREB (green), SOX2 (red, SCs marker), and DAPI (blue, nuclear marker) in organ of Corti explants treated with CDDP with or without FSK for 24 h.

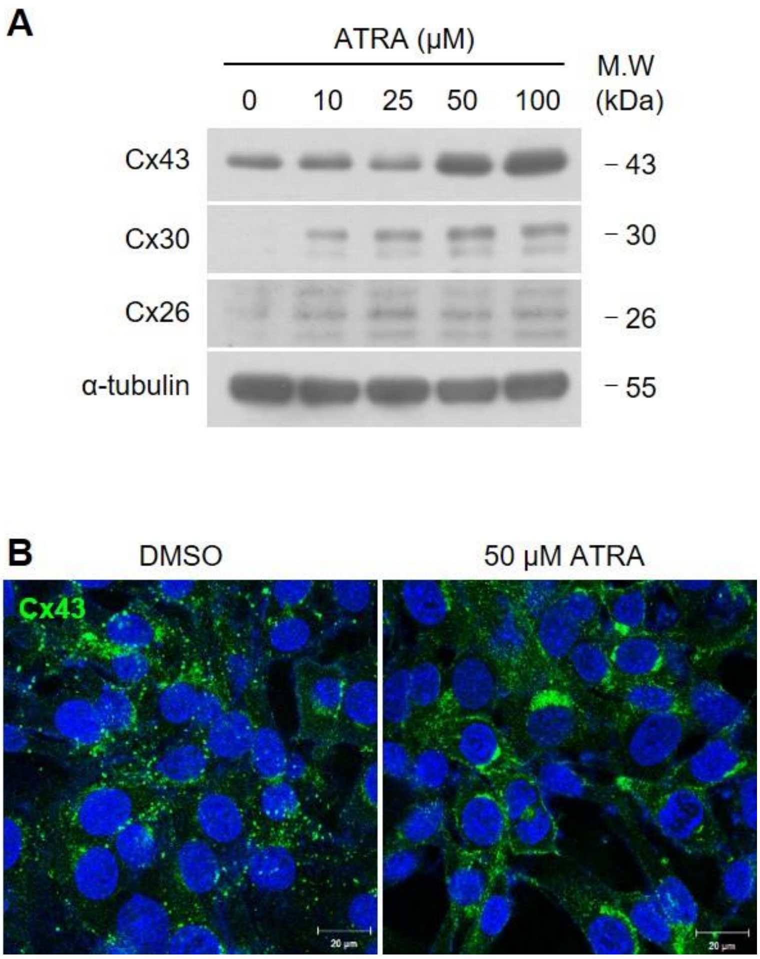

Supplementary Figure 2. ATRA up-regulates the expression of Cx43 and enhances GJIC in auditory cells. (**A**) HEI-OC1 cells were treated with different concentration (0-100 μM) of ATRA for 12 h. The Cx43, Cx30 and Cx26 detected by western blot. Proteins was normalized to α-tubulin levels. (**B**) Representative confocal images of Cx43 (green) and DAPI (blue) in the HEI-OC1 cells treated with DMSO or 50 μM ATRA. Scale bar=20 μm.

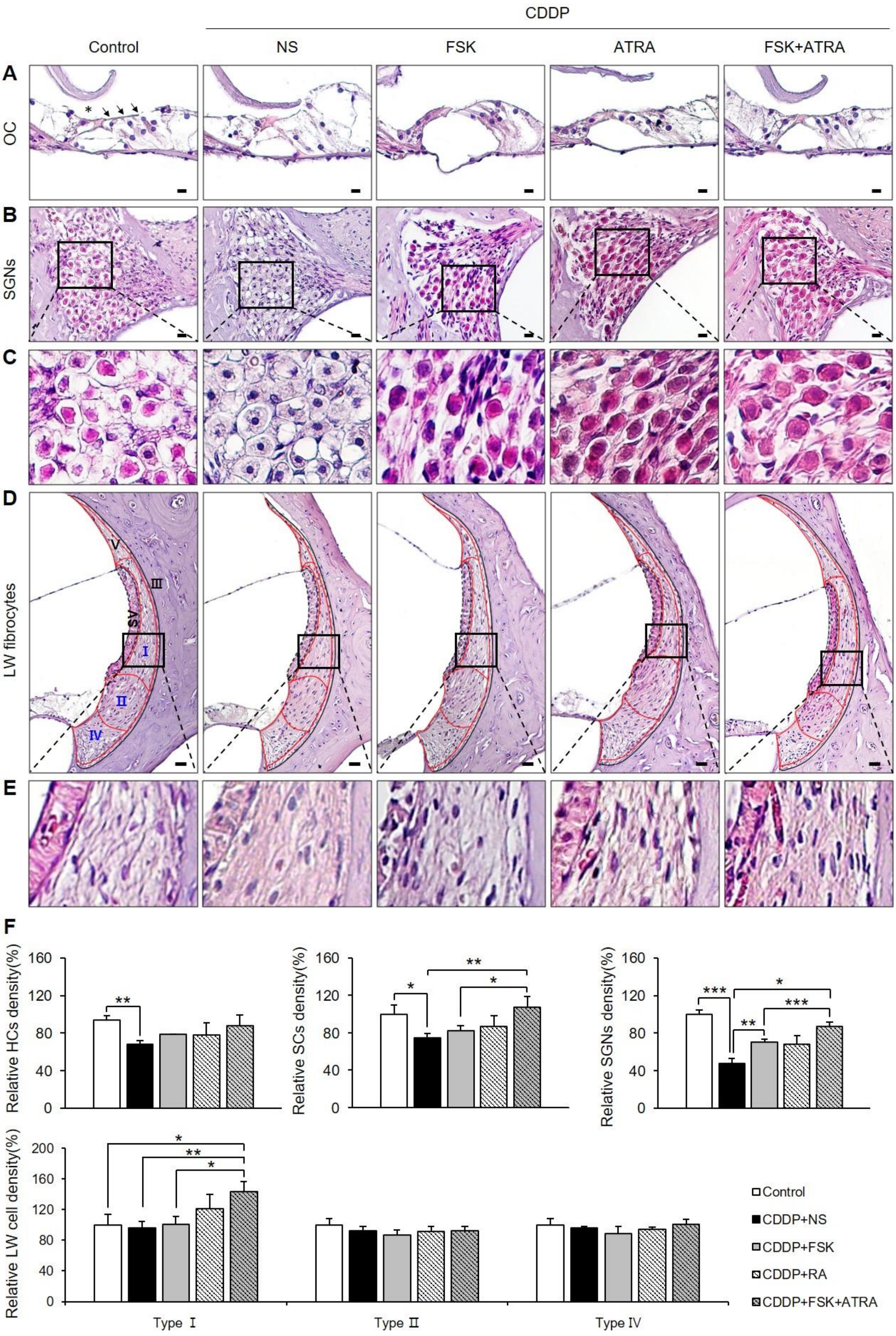
CDDP
Control
NS
FSK
ATRA
FSK+ATRA
A
OC
B
SGNs
C
D
LW fibrocytes
V
III
SV
I
II
IV
E
F
Relative HCs density(%)
Relative SCs density(%)
Relative SGNs density(%)
Relative LW cell density(%)
Type Ⅰ
Type Ⅱ
Type Ⅳ
Control
CDDP+NS
CDDP+FSK
CDDP+RA
CDDP+FSK+ATRA

Supplementary Figure 3. Histopathological evaluation of cochlea in rats treated with NS, FSK, ATRA and combination against CDDP-induced ototoxicity. Representative microphotographs of hematoxylin and eosin (H&E)-stained section, showing the OC **(A)**, SGNs (**B, C**) and LW fibrocytes (**D, E**) from middle turns of the cochlea (n=5 per groups). The square boxes in the image (**B, D**) indicate the approximate location of the high-magnification images illustrated in (**C, E**). Asterisk and arrows indicate IHC and OHC, respectively. IHC and OHCs are surrounded by different types of SCs in the OC. The area of each type of fibrocytes in the LW is divided by red line (**D**). (**F**) The bar graph represents the percentage of HCs, SGNs and LW fibrocytes at middle turns of cochlea for each group. Scale bar = 20 μm (A, B), Scale bar = 50 μm (D). $^{*}P < 0.05$, $^{**}P < 0.01$, $^{**}P < 0.001$. NS, Normal saline; CDDP, cisplatin; FSK, forskolin; ATRA, all-trans retinoic acid; OC, organ of Corti; SGNs, spiral ganglion neurons; SV, stria vascularis; LW, lateral wall.

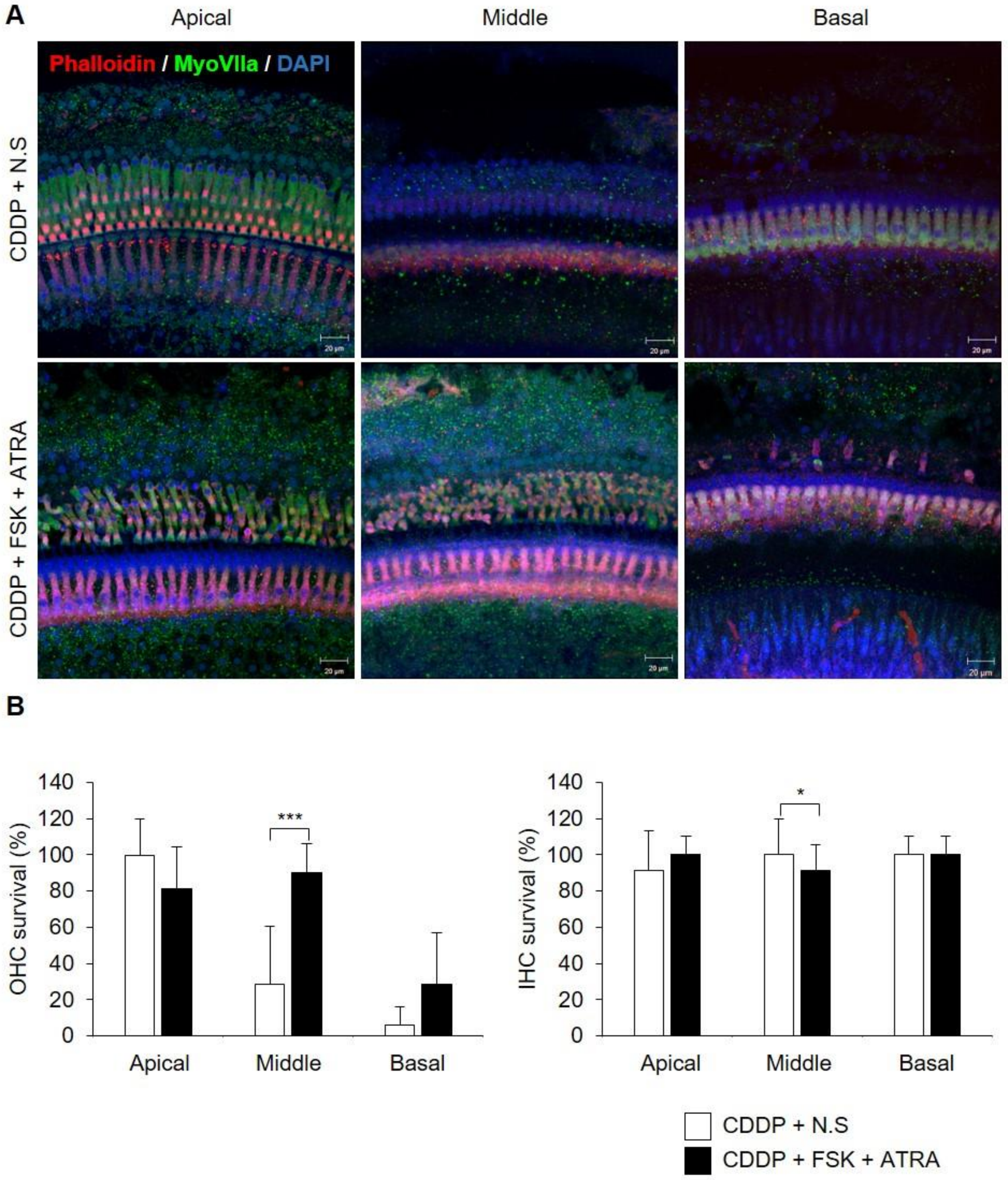

Supplementary Figure 4. FSK and ATRA combined treatment prevents HCs from CDDP-induced cell death. (**A**) Confocal images of phalloidin (red), MyoVIIa (green), and DAPI (blue) triple-labeled cells from the apex to the base in OC whole mount treated with N.S or FSK and ATRA in response to CDDP. Scale bar=20 μm. (**B**) Quantification of the OHCs and IHCs density in the apex, mid and base of OC whole mount. Data are presented as mean ± S.D, and statistically significant differences were identified using T-test. *$P < 0.05$, **$P < 0.01$, ***$P < 0.001$

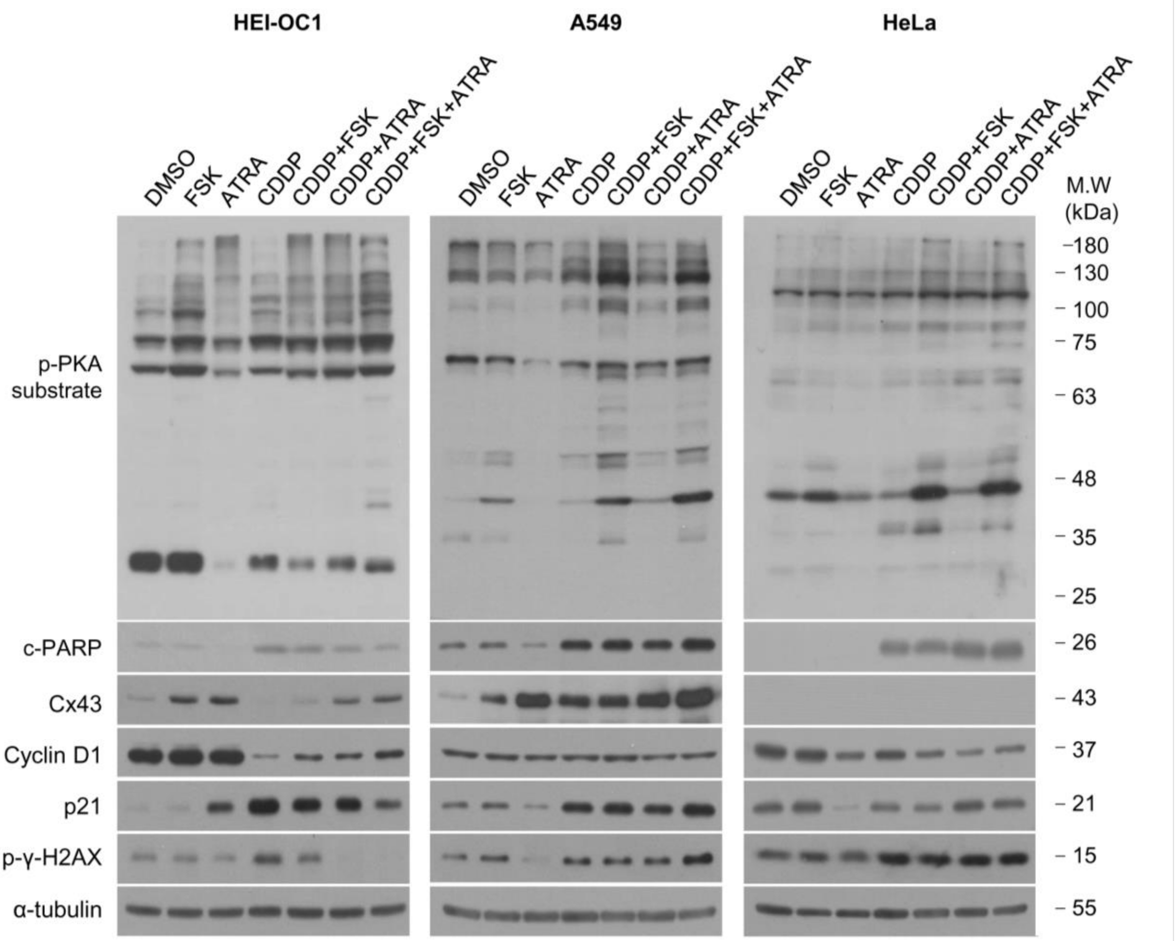

Supplementary Figure 5. Comparison of protein expression in auditory cells and cancer cells with FSK, ATRA, and combined treatments. Cells were pre-treated with 25 μM FSK, 50 μM ATRA, or a combination thereof for 2 h and subsequently treated with or without 25 μM CDDP for 24 h. Representative western blot results for p-PKA, c-PARP, Cx43, cyclin D1, p21, and phospho-γ-H2AX in HEI-OC1, A549, and HeLa cells. Equal protein loading was verified using α-tubulin.
